# Detecting mtDNA Effects with an Extended Pedigree Model: An Analysis of Statistical Power and Estimation Bias

**DOI:** 10.1007/s10519-025-10225-1

**Published:** 2025-07-16

**Authors:** Xuanyu Lyu, Michael D. Hunter, S. Alexandra Burt, Rachel Good, Sarah L. Carroll, S. Mason Garrison

**Affiliations:** 1 Institute for Behavioral Genetics, Department of Psychology and Neuroscience, University of Colorado at Boulder, Boulder, CO USA; 2 Pennsylvania State University, University Park, USA; 3 Michigan State University, East Lansing, USA; 4 Wake Forest University, Winston-Salem, USA

**Keywords:** Mitochondrial DNA, Extended family design, Structural equation modelling, Statistical power, Estimation bias

## Abstract

Mitochondrial DNA (mtDNA) plays a crucial role in numerous cellular processes, yet its impact on human complex behavior remains underexplored. The current paper proposes a novel covariance structure model with seven parameters to specifically isolate and quantify mtDNA effects on human complex traits. This approach uses extended pedigrees to obtain estimates of mtDNA variance while controlling for other genetic and environmental influences. Our Monte-Carlo simulations indicate that a sample size of approximately 5,000 individuals is sufficient to detect medium mtDNA effects ($mt^2 = 5\%$), while a more substantial cohort of around 30,000 is required for small effects ($mt^2 = 1\%$). We show that deeper pedigrees increase power to detect the mtDNA effect while wider pedigrees decrease power, given the equal total sample size. We evaluated how missing kinship records and mtDNA mutations impact bias. Both lead to underestimation of mtDNA variance, and an overestimation of the interaction between nuclear DNA and mtDNA. In addition, the false positive rate of mtDNA effect estimation is low when fitting the model with data generated without mtDNA effects. Collectively, we demonstrate that using extended pedigrees to quantify the influence of mtDNA on human behavior is robust and powerful.

## Introduction

### MtDNA Effect Estimation in Extended Pedigrees

Mitochondrial DNA (mtDNA) has garnered significant attention due to its unique characteristics and potential impact on human health. Unlike nuclear DNA, which undergoes recombination and is inherited from both parents, mtDNA reproduces asexually and is transmitted exclusively through the maternal line without recombination (Giles et al. 1980). Despite its small size, mtDNA plays an important role in cellular energy metabolism, implicating its dysfunctions in metabolic and degenerative disorders (Lin and Beal 2006; Lima et al. 2022). This distinct mode of inheritance allows mtDNA to pass unchanged from mother to offspring, making it a critical marker for genetic studies (as well as forensic identification). Although mtDNA has been implicated in a variety of aging-related and neurodegenerative disorders, including Alzheimer’s disease (Cha et al. 2012; Reddy 2009), Parkinson’s disease (Jin et al. 2014; Winklhofer and Haass 2010), Amyotrophic lateral sclerosis (ALS; Israelson et al. 2010; Murakami et al. 2007), and Huntington’s disease (Ayala-Peña 2013; Oliveira and Lightowlers 2010), a comprehensive and empirically validated model has yet to link mtDNA expression to age-related disorders (Cha et al. 2015; Lin and Beal 2006; Burt 2023).

The singular inheritance pattern of mtDNA complicates the detection of its effects using traditional behavior genetic methods. Behavior genetic models were developed for nuclear DNA, resulting in these models being ill-equipped to separate the effects of mtDNA from nuclear DNA. When such designs are employed, mtDNA is perfectly confounded with the shared environment because siblings in these designs (full siblings, identical twins, etc.) share the exact same mtDNA. This gap highlights the need for innovative approaches that can isolate mtDNA’s influence on phenotypic traits effectively.

**Fig. 1:**
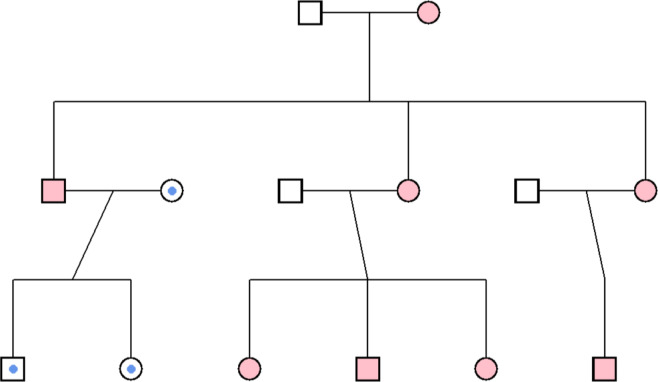
Example pedigree illustrating mtDNA inheritance generated using the ggpedigree R package. Squares denote male family members, and circles denote female members. Two distinct mtDNA lineages are shown, differentiated by both internal shape and shading (blue or pink). Individuals with the same combination of these features theoretically share identical mtDNA

To address these challenges, we developed a novel covariance structure model to quantify the impact of mtDNA differences on individual differences in human traits, such as aging-related diseases (Burt 2023; Burt et al. 2024). This model is designed to estimate mtDNA contributions by comparing phenotypic covariance among matrilineal-linked relatives to those without an unbroken chain of maternal relatives within large family datasets, while controlling for classic factors such as additive genetic variance and environmental variance. As all relatives on the matrilineal line in a family share 100% of their mtDNA and relatives on the patrilineal share 0%, we posit that the increased similarity of matrilineal relatives indexes mtDNA effects on the outcome (see Fig. 1 for a visual demonstration; Garrison 2025). Statistically, the covariance structure model estimates multiple sources of influence on the variance of outcomes:

1 $$\begin{aligned} {\textbf {Cov}}(\varvec{\vec {p}})= & ({\textbf {A}} \cdot a^2) + (\mathbf {I_{AA}} \cdot i^2) + ({\textbf {C}}_{\textbf {F}} \cdot c_F^2) + ({\textbf {C}}_{\textbf {E}} \cdot c_E^2) \nonumber \\ & + ({\textbf {M}} \cdot mt^2) + ({\textbf {J}} \cdot j^2) + ({\textbf {E}} \cdot e^2) \end{aligned}$$

In Eq. 1, ${\textbf {Cov}}(\varvec{\vec {p}})$ is the estimated ${N \times N}$ phenotypic outcome covariance matrix, where $\textit{N}$ is the number of people who have at least one non-zero relationship with any other person. The uppercase letters denote relatedness matrices, which are known and fixed in the data structure and indicate the degree of relationship between two individuals. The lower-case letters are path coefficients regressing latent variance components on the observed phenotypic variable. After being squared, these letters indicate standardized variance components that are freely estimated. ${\textbf {A}}$ stands for additive nuclear DNA relatedness matrix. The ${a^2}$ coefficient represents the estimated amount of variance associated with the additive genetic relatedness pattern.^1^$\mathbf {I_{AA}}$ stands for a two-allele nuclear DNA epistasis relatedness matrix. The relatedness coefficient in this matrix represents the joint probability distribution of sharing two independent alleles between two individuals. This matrix is calculated by element-wise squaring the additive genetic matrix $\textbf{A}$; equivalently, each entry is computed by multiplying the corresponding values from two copies of $\textbf{A}$. The variance component associated with this matrix is represented as $i^2$.

The mitochondrial relatedness matrix is abbreviated as ${\textbf {M}}$, and its corresponding variance is ${mt^2}$. For kin pairs with shared maternal ancestry, it will be indicated as one, and zero otherwise. ${\textbf {J}}$ stands for the interrelated regulation between nuclear and mitochondria, which is calculated by element-wise multiplying the corresponding matrix entries in ${\textbf {A}}$ and ${\textbf {M}}$. For any pedigrees that include pairs of individuals who share mitochondrial genomes (M relatedness = 1) but differ in nuclear relatedness (A relatedness = 0.125 versus 0.25) as well as pairs who share only nuclear genomes (M = 0) at the same A = 0.125 level, the product term ${\textbf {J}}$ (the interaction of additive and mitochondrial relatedness) varies independently of both A and MT alone. In practice, having at least two dyads with MT = 1 but different A-and at least one dyad with MT = 0 at that same A-means the covariance attributable to ${\textbf {J}}$ cannot be confounded with the main additive or mitochondrial components.

**Fig. 2:**
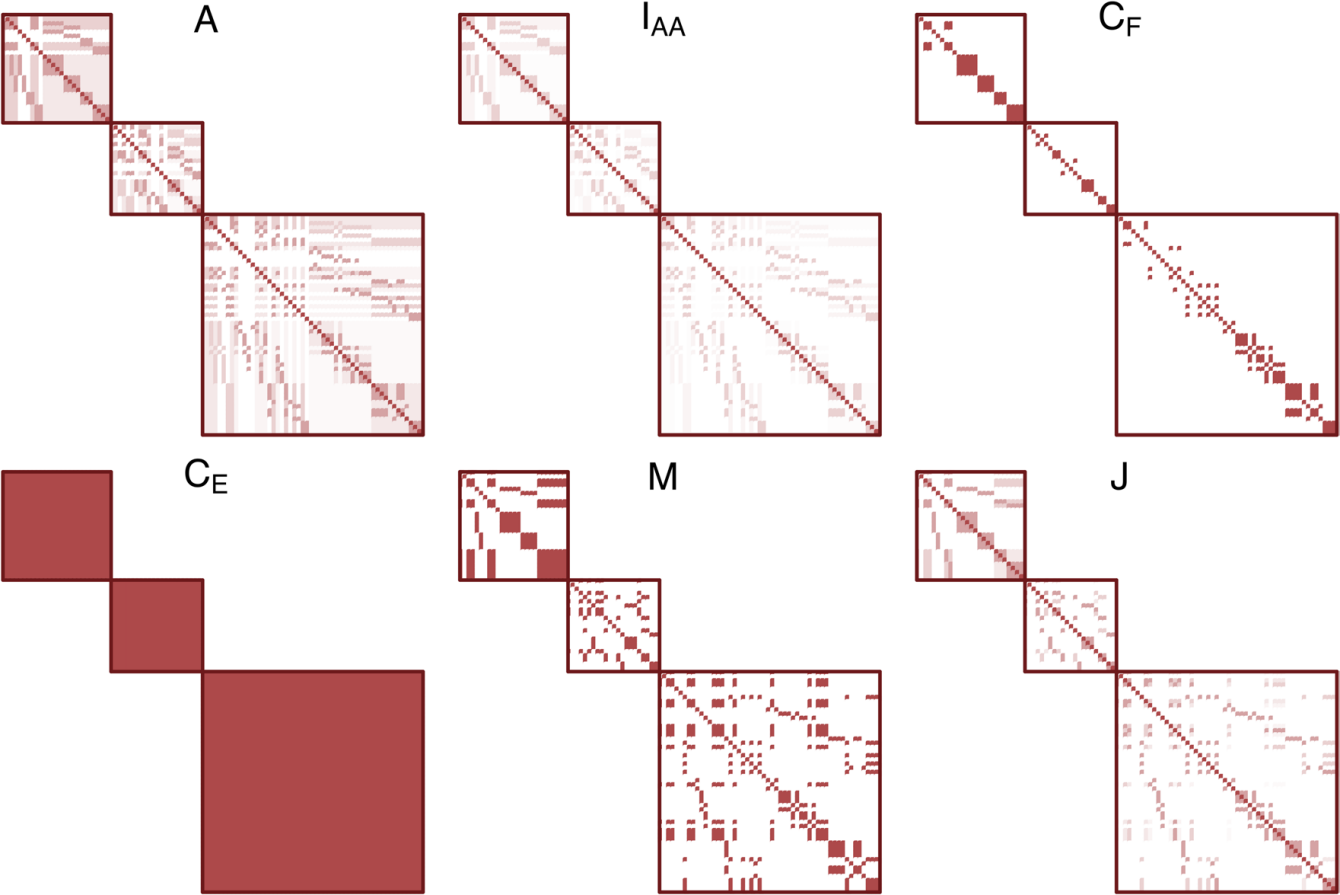
Patterns of relatedness matrices in Eq. 1, derived from three simulated pedigree structures. Darker cells indicate values closer to 1. Lighter cells indicate values closer to 0

$\varvec{C_F}$ indicates the matrix of common nuclear family environment similarity. It will be coded as one if the members share the same mother and father. $\varvec{C_E}$ is the similarity due to the common extended family, where every member in the same pedigree^2^ will be coded as one. ${\textbf {E}}$ is the unique environment matrix and random error (Burt 2023). Figure 2 illustrates the pattern of all relatedness matrices using three random pedigrees.

The proposed model represents a pioneering approach to quantifying the impact of mtDNA on human traits. Multiple biological pathways have been found to link mtDNA differences with phenotypical differences (Ferreira and Rodriguez 2024). The primary effects come from the transcription and translation of the 37 mtDNA encoded genes (Anderson et al. 1981). The encoded genes are translated into rRNA, tRNA and polypeptides that serve the important functions of energy production through Complex I to V and cell cycle regulations through mitochondrial-derived peptides (Moraes et al. 2002; Gruschus et al. 2023). Another important region of mtDNA is the non-coding region and the displacement loop within the non-coding region, which regulates mtDNA transcription and produces vital proteins for oxidative phosphorylation (Nicholls and Minczuk 2014; Ferreira and Rodriguez 2024). Both processes mentioned above contribute to the phenotypic variance that can be captured by the $mt^2$ component of our model. One other biological property of mtDNA is that the number of copies of mtDNA within a cell differs between different individuals (Guyatt et al. 2019). There has been some preliminary research suggesting that the number of copies of mtDNA is associated with several molecular processes and human diseases such as dementia (Castellani et al. 2020; Chong et al. 2022). Both twin analysis and GWAS suggest that the copy number of mtDNA is regulated by nuclear DNA and influenced by environmental factors (Xing et al. 2008; Chong et al. 2022). Therefore, we expect the individual differences caused by the difference in copy number of mtDNA to be accounted for under $a^2$, $i^2$, $c_F^2$, $c_E^2$ and $e^2$.

One advantage of the current model is its ability to estimate mtDNA effects separately from the well-studied influences of nuclear-genetic and environmental factors. In addition to yielding mtDNA estimates that are not masked by other factors, the current model can also be used to estimate the nuclear-genetic and environmental effects that are not contaminated by mitochondrial influences. This framework harnesses large pedigrees to support simultaneous estimation of multiple variance components. The path tracing rules for deriving the relatedness matrices provide a solid foundation for leveraging unlimited amounts of kin pairs in pedigrees of various sizes, from small nuclear family pedigrees to pedigrees with more than 750,000 individuals (Burt et al. 2024). Previous family designs have been limited to nuclear families and extended twin designs, which have hindered researchers’ ability to estimate more variables due to a lack of degrees of freedom from additional pairwise kinship in extended pedigrees (Hunter et al. 2021).

Although mathematically and theoretically promising, the data used for fitting such a model faces stringent requirements. To obtain estimates of mtDNA effects, the data require family pedigrees with various unique relatedness patterns on the seven parameters. Furthermore, the family data should include a record of relevant individual outcomes. For example, since many neurodegenerative diseases manifest in relatively late periods of life, data must span decades for the disease to theoretically manifest in all involved generations.

### Statistical Power of MtDNA Effects Estimation

Statistical power is the probability of correctly rejecting the null hypothesis (Cohen 1988, 1992). As the extended pedigree model is a covariance structure model, its power analysis follows the general framework of power analysis for structural equation modeling (SEM). To test the significance of a specific variance component (e.g., $mt^2$) in a covariance-based structural equation model, the null hypothesis is that the best-fit covariance structure is the one without the specific variance component (e.g., a model without $mt^2$). The alternative hypothesis is that the best-fit covariance structure is the one with the specific variance component (e.g., a model with $mt^2$; Satorra and Saris 1985). Complex covariance-based structure models are commonly fit with maximum likelihood estimation (Rao et al. 1987), which primarily employs the likelihood ratio test for hypothesis testing (Curran et al. 2002; Jobst et al. 2021; MacCallum et al. 1996; Satorra and Saris 1985). Thus, the power to detect the estimated parameter is essentially the power of the likelihood ratio test when comparing the model under the alternative hypothesis against the model under the null hypothesis (Satorra and Saris 1985). If the difference in log-likelihood ratio between the two models is statistically significant, we will regard that the model constructed from the alternative hypothesis fits significantly better than the model constructed from the null hypothesis.

Given the small scale of mtDNA relative to nuclear DNA (16.6k base pairs of complementary nucleotide versus 2,875,002k base pairs of complementary nucleotide; Marande and Burger 2007), a safe assumption to be made is that the effect size of mtDNA will be substantially smaller than that of nuclear DNA for most complex traits. Previous studies have observed unusually large shared genetic influence for heights between maternally-linked cousins, which may be attributed to the impact of mtDNA (Garrison et al. 2018; Rodgers et al. 2019), but there are no previous studies directly quantifying overall mtDNA effects. Moreover, the existence of mtDNA effects is likely to vary from outcome to outcome. Hence, a power analysis should be tailored and conducted to evaluate the sample size requirement under different conditions (e.g., various family structures; different sets of estimated parameters; different effect sizes of the mtDNA effects).

Since the current covariance structure model is an extension of a classical twin-family ACE model, factors that impact the power of the ACE model could serve as a heuristic starting point for the current model. Previous studies have thoroughly discussed how sample size (Martin et al. 1978; M. Neale and Cardon 2003; Verhulst 2017; Visscher 2004), variance components (Verhulst 2017), the type-one error rate (Sham et al. 2020), and the ratio of twin types (Visscher 2004; Visscher et al. 2008; Lyu and Garrison 2023) impact the power of parameter estimation in the ACE model. In the case of mtDNA effects, it is reasonable to hypothesize that some properties of the family structure will impact the power estimation, given the fact that the power of the ACE model is also impacted by the ratio of MZ and DZ twins and the distribution of variance contribution (Martin et al. 1978; Visscher 2004). Visscher 2004 found in every variance combination of the ACE model, there exists an optimal ratio of MZ over DZ for a given sample size to reach a specific power. For example, when the population variance components are distributed as $a^2$ =.6, $c^2$ =.1, to achieve a power of.95, a total of 235 pairs of twins is required, of which 70% should be MZ twins. For the mtDNA effect, power can be potentially impacted by the proportion of matrilineal relatives (relatives who share the identical mtDNA in a family) and patrilineal relatives (relatives who do not share the same mtDNA in a family). Just as there exists an optimal ratio for MZ and DZ twins in ACE model estimation, an optimal ratio between the matrilineal and patrilineal relatives should exist for mtDNA effect estimation. However, in reality, researchers have very limited control of the sex^3^ distribution in pedigree datasets. In all our simulations, we fixed the sex ratio at biological male: biological female at 1:1.

Families with different structures inherently generate different relatedness matrices, which influence model estimation. Including additional unique relatedness matrices in the model increases the degrees of freedom for parameter estimation, thereby enhancing statistical power, as demonstrated in previous research on covariance-based structural equation modeling (MacCallum et al. 1996). However, for a given number of individuals, larger families reduce the number of distinct families included in the analysis. This, in turn, limits the variety of relatedness matrices available for estimation. Several properties of a pedigree, such as the number of sibships per family and the number of generations included, shape the structure and scale of each relatedness matrix that can be derived (Chen and Abecasis 2006; Hunter et al. 2024). Conducting systematic research on how pedigree structures impact mtDNA effect estimation power will provide useful guidelines for future users of the current model. It will contribute to the field’s understanding of the incorporation of extended kinships beyond siblings and nuclear families, as such designs have demonstrated effectiveness in reducing biases inherent in twin/sibling studies and in offering fresh perspectives (Keller et al. 2010).

The shape of a pedigree is primarily determined by the number of generations included (the depth of a pedigree) and the average number of offspring per mate (the width of a pedigree). Additional factors, such as the proportion of individuals who reproduce (defined here as the reproductive rate) and the ratio of male to female offspring (newborn sex ratio), also influence the structure of the pedigree. Although these factors are important and can have implications in estimating measures of reproductive fitness (Borger et al. 2023), our analysis focuses on operationalizing the shape of the pedigree by varying the number of generations and the average number of offspring per couple.

### Model Assumptions and Estimation Bias

Like other family structures, unbiased estimation of mtDNA effects relies on ensuring certain assumptions to avoid model misspecification. For instance, in classical twin studies, the model’s robustness depends on assumptions such as random mating, which validates the 0.5 genetic relatedness in dizygotic twins, and the equal environment assumption, indicating that both MZ twins and DZ twins share family environments to the same extent (Felson 2014; Neale and Cardon 2003). However, applying the extended pedigree model introduces several challenges:

1. Firstly, the estimation can be impacted by unobserved kinship in large datasets, as estimating mtDNA effects requires extended pedigrees. For example, if two nuclear families are connected through the same mother, but one family lacks documentation of this maternal relationship in the dataset, the mtDNA relatedness matrix will inaccurately contain zeros where there should be ones. Such missing links can potentially affect the accuracy of mtDNA effect estimation. No family dataset is collected perfectly, and all of them have unidentified or incorrectly identified kinship links to some extent, depending on the data collection practice. Hence, testing the robustness of the estimation given an imperfect kinship record ensures the efficacy of the model as a pivoting method for detecting mtDNA effects.
2. Secondly, the current model assumes no mutation of mtDNA across generations. Like nuclear DNA, mtDNA mutates randomly during replication and the stability of polynucleotide chains decreases under certain external influences (DiMauro and Schon 2001). Given that mtDNA comprises only about 17k base pairs and 37 encoding genes, any mutation could lead to a misspecification of mtDNA relationships among individuals sharing the same matrilineal line in a pedigree. However, in most datasets where this model will potentially be applied, actual mtDNA sequencing is unlikely to have been performed. Given a mutation rate of $2.7 \times 10^{-7}$ per site per generation, we estimate that the expected number of mutations per generation is around $4.59 \times 10^{-3}$ in germline cells. In other words, the mutation rate suggests that one mutation is expected to occur every 218 parent-offspring transmissions.^4^ Although the absolute mutation rate is low, if an undetected mutation exists between the early generations of the pedigree, the mtDNA relatedness matrix will be misspecified for many descendants after the mutation. Hence, understanding how the estimates of $mt^2$ will be impacted by this limitation is important for future applications.
3. Thirdly, unlike the nearly universal additive genetic effects observed across human traits, mtDNA’s influence may be minimal for certain traits. Therefore, assessing the false positive rate of the estimation is crucial to ensure that variances from other components are not mistakenly attributed to mtDNA effects, when there are no mtDNA effects for the specific traits.

## Methods

In this study, we employed Monte Carlo simulations to investigate two critical statistical properties of the extended pedigree model. First, we examined how statistical power for mitochondrial DNA (mtDNA) estimation varies across different sample sizes and explored the influence of diverse pedigree structures on power. Second, we evaluated the effects of specific model assumption violations on estimation bias, quantifying both the direction and magnitude of the bias under each scenario. All simulation R code for this paper is available at https://github.com/Xuanyu-Lyu/mtDNA, along with the accompanying R package (BGmisc; Garrison et al. 2024, 2025).

### Power Analysis

In the current analysis, we operationalize the power of mtDNA estimation as the power to detect $mt^2$ and $j^2$ simultaneously, as both variances are vital components of the mtDNA effects. In the current study, our null ($H_0$) and alternative hypothesis ($H_1$) are:

$H_0$: The mtDNA ($mt^2$) and its interaction with nuclear DNA ($j^2$) do not have a non-zero effect on a specific phenotype.

$H_1$: The mtDNA ($mt^2$) and its interaction with nuclear DNA ($j^2$) have a non-zero effect on a specific phenotype.

To evaluate the significance of the likelihood ratio test, a unit test statistic (*T*) is computed by comparing the fits of two models: the null hypothesis model and the alternative model. The test statistic is calculated as the difference in their respective negative twice-log-likelihood values, normalized by the total sample size (*N*) used in the simulation:

2 $$\begin{aligned} T = \frac{-2ll(H_1) - (-2ll(H_0))}{N} \end{aligned}$$

here, $ll(H_0)$ and $ll(H_1)$ represent the negative twice-log-likelihood values derived from fitting the null hypothesis model and the alternative model, respectively, while *N* denotes the total number of individuals included in the simulation.

The test statistic $T$ follows a non-central chi-square distribution:

3 $$\begin{aligned} T \sim \chi ^2_{2}( \lambda ) \end{aligned}$$

where 2 is the degrees of freedom of the distribution and the non-centrality parameter ($\lambda$) is derived from

4 $$\begin{aligned} \lambda = (N'-1)T \end{aligned}$$

where $N'$ is the target sample size that we want to derive power from.

Then, the statistical power of the likelihood ratio test given any sample sizes can be derived by comparing the central $\chi ^2$ distribution from the null hypothesis and the non-central $\chi ^2$ distribution from the alternative hypothesis:

5 $$\begin{aligned} \text {Power} = P(\chi ^2_{2} (\lambda ) > \chi ^2_{2, \alpha }(0)) \end{aligned}$$

where $\chi ^2_{2} (\lambda )$ is a random variable following a non-central chi-square distribution with 2 degrees of freedom and non-centrality parameter $\lambda$, and $\chi ^2_{2, \alpha }(0)$ is the critical value from the central chi-square distribution at significance level $\alpha$. In the current study, $\alpha =.05$.

One convenient property of power analysis for the likelihood ratio test is that only one robust unit test statistic *T*, which is the contribution to $\lambda$ from one individual, is required. Afterward, power for any sample sizes under the same conditions can easily be derived numerically using Eqs. (4 and 5). The essential unit test statistic (*T*) for different pedigree structures is obtained by averaging *T* observed from 100 simulation sets, each including 10,000 individuals. The association between sample sizes and power for various conditions can be easily derived when an accurate non-centrality chi-square parameter is obtained through simulation (Satorra and Saris 1985; Verhulst 2017).

For family pedigrees, various factors such as the number of generations involved, the average number of offspring per mate, sex ratios of newborns, reproductive rates within a generation, inbreeding, remarriage rates, and adoption rates significantly influence the structure and size of the family pedigree. As noted, the depth (number of involved generations) and the width (average offspring per couple) of a pedigree are also critical in shaping the overall family structure and determining the total family size. To investigate the impact of these two factors, we set up five conditions of parameters that influence family structure (Table 1). Among these conditions, pedigrees 1, 2, and 3 are designed for evaluating the impact of the number of generations, and pedigrees 1, 4, and 5 are designed for evaluating the impact of offspring per mate. Due to the relatively low prevalence of factors like consanguinity and twins, we did not perform power analysis under variation of other pedigree structure parameters. We did not analyze the impact of other factors including sex ratios and reproductive rates pedigrees on power, since their impact is rather limited in a real-life setting, and researchers have very little control over these factors. We fixed sex ratios of newborns at 1:1 and the reproductive rates at.7. We also assumed that these low prevalence scenarios, such as consanguinity and twins, did not exist in our pedigrees to reduce design complexity and increase computational efficiency.

**Fig. 3:**
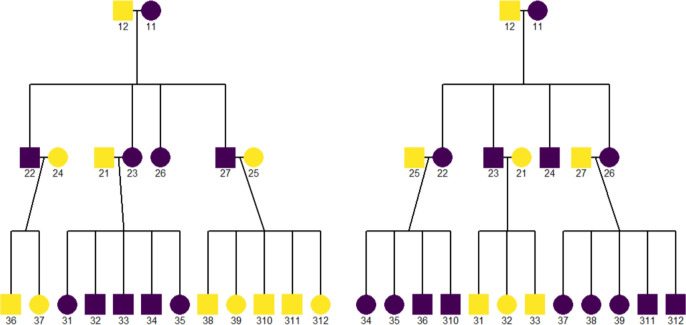
Two pedigrees generated with the same parameters: number of generations (*G*) is 3, number of offspring per mate (*k*) is 4, male offspring proportion (*p*) is 0.5, and reproductive rate (*r*) is 0.7, using the *simulatePedigree()* function from the BGmisc R package

Family size is another important factor in family structure. In the current study, we did not set family size as a self-varied parameter. The family size of one specific condition is a weighted exponential function of the number of generations, average offspring, and reproductive rate under the current assumptions of family structure. In Eq. 6, for any $g \in \mathbb {Z}$

6 $$\begin{aligned} m_g = f(g) = {\left\{ \begin{array}{ll} 2, & g = 1 \\ k^{g-1}r^{g-2}(1 + r), & 2 \le g \le G - 1 \\ k^{g-1}r^{g-2}, & g = G \end{array}\right. } \end{aligned}$$

where $m_g$ is the number of individuals in the $g^{th}$ generation, *k* is the number of offspring per mate, *r* is the reproductive rate, and *G* is the total number of generations. The number of individuals *m* in a pedigree with *G* generations can be calculated with $m = \sum _{g=1}^{G} m_g$. For example of a pedigree in the left panel of Fig. 3 the second generation has $k=4$, $r =.7$ and $g = 2$. Therefore, the second generation will have a total number of around $4^1 \times 0.7^0 \times (1+.7) \approx 7$ individuals.

**Table 1:** Pedigree structures used in simulation

Pedigree	k	G	p	r	m
1	3	4	0.5	0.7	33
2	4	4	0.5	0.7	61
3	8	4	0.5	0.7	344
4	3	6	0.5	0.7	388
5	3	8	0.5	0.7	657

*k* number of offspring per mate, *G* number of generations, *p* male offspring proportion, *r* reproductive rate, *m* pedigree size

We developed a simulation function that generates random pedigree structures based on user-defined parameters, ensuring variability in the simulated data to more closely resemble real-world scenarios (Garrison et al. 2024). In this model, the number of offspring for a given pairing is determined by a Poisson distribution, where the mean is set to the user-specified average number of offspring. This approach allows for variation within each generation, although the total number of generations remains constant, as dictated by the user’s parameters. The exact number of generations in a simulated pedigree is fixed at the value of the user-specified parameter for the convenience of deriving the non-centrality parameter, as ideally, the size of the pedigree should remain constant. All simulated pedigrees will start with an identical 1st generation that only has one mated couple. The pedigree will end with a generation in which no individual has mated. For instance, Fig. 3 presents two example pedigree plots from the same parameter setting ($G = 3$, $k = 4$, $r =.7$) of the simulation function. Both pedigrees have four generations, but the number of offspring across mates are different, while the size of the pedigree remains almost identical.

In our simulations, we introduce four distinct levels of $mt^2$ estimates to explore the sample sizes necessary to detect various levels of mtDNA effects. The specified variance levels explained by mtDNA are set at 0.5%, 1%, 5%, and 10%. Specifically, we define $a^2 =.40$, $i^2 = 0$, $c_F^2 =.10$, $c_E^2 =.10$, and $j^2 =.01$. As established by previous research on ACE models (Visscher 2004; Verhulst 2017), the estimation of any given variance component can be influenced by the magnitudes of other components within the same model. Consequently, the statistical power to detect variance attributable to mitochondrial DNA ($mt^2$ and $j^2$) is likely affected by the levels of additive genetic variance ($a^2$) and shared nuclear family variance ($c_F^2$). To explore these interdependencies, we conducted simulations by systematically varying three levels of additive genetic variance ($a^2 = 0.2, 0.3, \text {or } 0.4$) and two levels of shared nuclear family variance ($c_F^2 = 0.2 \text { or } 0.3$), while the true $mt^2$ was fixed at 0.01.

### Estimation Bias When Violating Assumptions

In the current study, we analyzed the estimation bias that arises when various model assumptions are not met. We used the BGmisc package (Garrison et al. 2024) to simulate data that did not meet the assumptions and then fit the model assuming that the assumptions were met. Our goal was to determine if the estimates of mtDNA effects remained robust even when the data did not meet the model assumptions, or when we were uncertain if the data met the assumptions. All simulations conducted to evaluate estimation bias use a variance combination of $a^2 = 0.40$, $i^2 = 0$, $mt^2 =.05$, $c_F^2 =.10$, $c_E^2 =.10$, and $j^2 =.01$.

The first potential source of estimation bias we examined was the assumption that the pedigree structure in the data is intact-an assumption that is unlikely to hold in most real-world pedigree datasets. Deviations from the true pedigree can be conceptualized as random noise in the pedigree structure. Incomplete sampling may separate relatives who, in reality, belong to the same pedigree. This is particularly consequential when estimating the effects of mtDNA, as maternal relatives may be omitted from kinship records. Such omissions may occur if a critical link, such as a partner or parent-offspring relationship, is missing from the true pedigree. As a result, some relative pairs may be misspecified as unrelated in all genetic and environmental relatedness matrices, potentially biasing estimates of mtDNA effects. To examine this bias, we simulated 500 sets of 124 pedigrees (*n* = 161; *k* = 3; *G* = 5; *r* = 1, yielding total sample size of *N* = 19964 per simulation). Phenotypic data were generated based on relatedness matrices derived from the true complete pedigree. We then introduced misspecification by randomly selecting a female relative in the second generation and removing her kinship links with her mother and father. We repeated the process for all 10,000 simulated pedigrees. The model was fitted using correct and incorrect relatedness matrices, resulting in two sets of estimates of mtDNA effects. By comparing the distribution of 500 estimates in both the correct model and the misspecified model, we can evaluate the bias introduced by unobserved maternal links in the pedigree.

The second source of potential bias we evaluated was the unmeasured presence of mtDNA mutations. If a female relative in a pedigree carries a mutation in her mtDNA when forming her gametes, this mutation will be inherited by all her maternal-line descendants. As a result, her descendants will have a mtDNA relatedness coefficient of 0 with all other relatives in the pedigree, who are not direct descendants, even though they share the same maternal lineage. In other words, the individual who carries the mtDNA mutation and all their maternal progeny will have a different haplotype of mtDNA from the individual’s maternal ancestors. This is consistent with how we designate shared and non-shared mtDNA haplogroups in the mtDNA relatedness matrix initially. In our model, we assume that all maternal relatives in a pedigree share the same mtDNA. However, this assumption is incorrect when a mutation occurs and will lead to a misspecified mtDNA relatedness matrix. The missing mutation can cause a biased estimation of mtDNA effects. We thus simulated 500 sets of 124 pedigrees (*n* = 161; *k* = 3; *G* = 5; *r* = 1, and total sample size of each simulation *N* = 19964). Then, we randomly selected a female relative in the 2nd generation and disconnected her from her parents. We generated the mtDNA relatedness matrix with the mutated pedigree, but all other relatedness matrices are generated from the intact pedigree. We examine the bias from not incorporating mutations by comparing the distribution of 500 estimates from the correct model (with mutation) and the model in which the mtDNA relatedness matrix is misspecified (without mutation).

Third, we investigate the impact of applying the full covariance structure model with seven parameters to phenotypes that are not influenced by mtDNA (i.e., $mt^2 = 0$, $j^2 = 0$). By fitting the full model with the simulated data, we can evaluate the proportion of the 500 estimates that have values exceeding a certain numerical value as a metric of the false positive rate of the $mt^2$ estimate. We simulated 200 sets of 124 pedigrees (*n* = 161; *k* = 3; *G* = 5; *r* = 1, and total sample size of each simulation *N* = 19964) and generated data using a covariance matrix without the contribution of mtDNA effects.

## Results

### Power

#### Power as a Function of Effect Sizes

In Table 2, we present the sample size required to reach a specific power level under various effect sizes of $mt^2$. To compare the effects of pedigree structure, we designated pedigree 4 as a large pedigree and pedigree 1 as a small pedigree. If not specified, we used a variance combination of $a^2 = 0.40$, $i^2 = 0$, $mt^2 =.05$, $c_F^2 =.10$, $c_E^2 =.10$, and $j^2 =.01$ for all other parameters. In general, 30,000 individuals will grant adequate power when 1% or more of the phenotypic variance is explained by mtDNA. However, larger sample sizes are required for traits under smaller mtDNA effects, especially when using smaller pedigrees.

**Table 2:** Power analysis for different pedigree sizes and variance levels

Pedigree size	Variance level	Power			
		0.4	0.6	0.8	1.0
Large pedigree (m = 388)	$mt^2$ =.1	576	933	1447	5478
	$mt^2$ =.05	1861	3017	4678	17708
	$mt^2$ =.01	11671	18919	29340	111073
	$mt^2$ =.005	14630	23717	36780	139240
Small pedigree (m = 33)	$mt^2$ =.1	607	984	1526	5775
	$mt^2$ =.05	2038	3304	5124	19396
	$mt^2$ =.01	12312	19959	30953	117179
	$mt^2$ =.005	13995	22687	35183	133193

The table presents the sample size (number of individuals) required for specific power levels to detect mtDNA variance at different $mt^2$ levels in large and small pedigrees

Table 3 presents how varying levels of variance attributable to additive genetic effects ($a^2$) and nuclear family effects ($c_F^2$) impact the sample size required to achieve a specified statistical power for estimating mtDNA effects ($mt^2$ and $am^2$). Generally, a larger magnitude of $a^2$ corresponds to a reduced sample size needed for robust detection of mtDNA effects at a given power level. In contrast, a larger magnitude of $c_F^2$ necessitates an increased sample size to maintain the same level of statistical power for these estimations.

**Table 3:** Required sample sizes for MtDNA estimation power at different $a^2$ and $c_F^2$ levels

$c_F^2$ level	$a^2$ level	Power			
		0.4	0.6	0.8	1.0
$c_F^2 =.2$	$a^2 =.2$	11873	19247	29848	112996
	$a^2 =.3$	11774	19086	29599	112053
	$a^2 =.4$	10618	17213	26694	101058
$c_F^2 =.3$	$a^2 =.2$	13617	22074	34232	129594

The table presents the sample size (number of individuals) required for specific power levels to detect mtDNA variance. The data correspond to a medium pedigree structure (m = 61) with a constant $mt^2$ level

#### Pedigree-Number of Generations

**Fig. 4:**
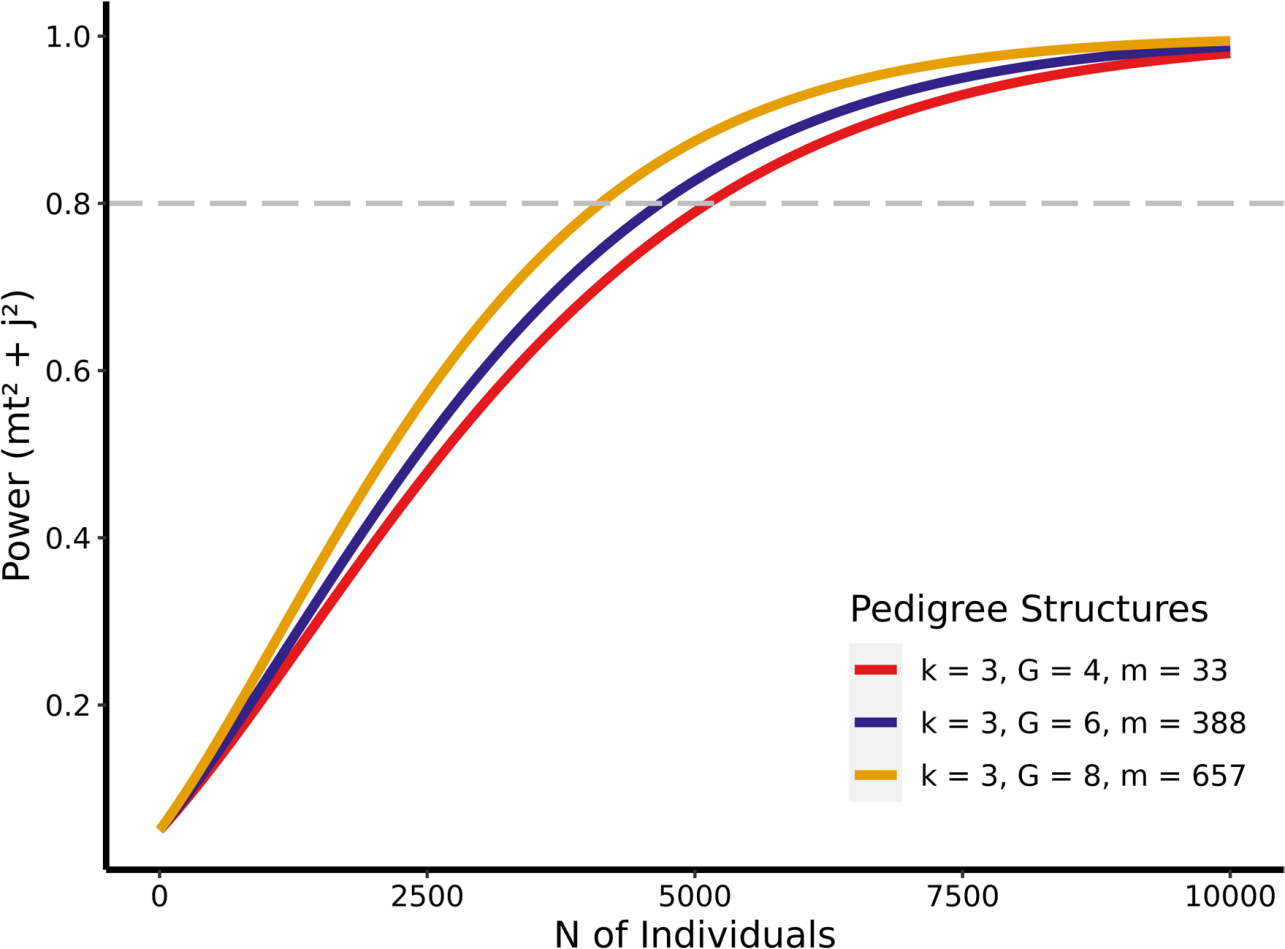
Estimation power for $Mt^2$ and $J^2$ as a function of total sample sizes and number of generations in simulated pedigrees. *k* number of offspring per mate, *G* number of generations, *m* pedigree size. Effect sizes of variance components: $a^2 = 0.40$, $i^2 = 0$, $mt^2 =.05$, $c_F^2 =.1$, $c_E^2 =.1$, and $j^2 =.01$

In Fig. 1, three pedigree structures with varying numbers of generations (*G*) are compared in terms of power to detect mtDNA effects. In these pedigrees, each mate has three offspring (*k* = 3). The findings from Fig. 4 demonstrated that pedigrees with a larger number of generations have increased power. When examining pedigrees with an eight-generation depth (*G* = 8), a power of.8 is attained with 4,011 individuals, utilizing a variance combination with medium mtDNA effects ($mt^2$ =.05, $j^2$ =.01). On average, across all simulated variance component combinations, using four-generation pedigrees requires 27.76% larger sample sizes compared to five-generation pedigrees in order to achieve a power of.8. Conversely, the smallest pedigrees (*G* = 4) exhibit the lowest power, necessitating a sample size of 5,124 individuals to achieve a power of 8.

#### Pedigree-Offspring Per Mate

**Fig. 5:**
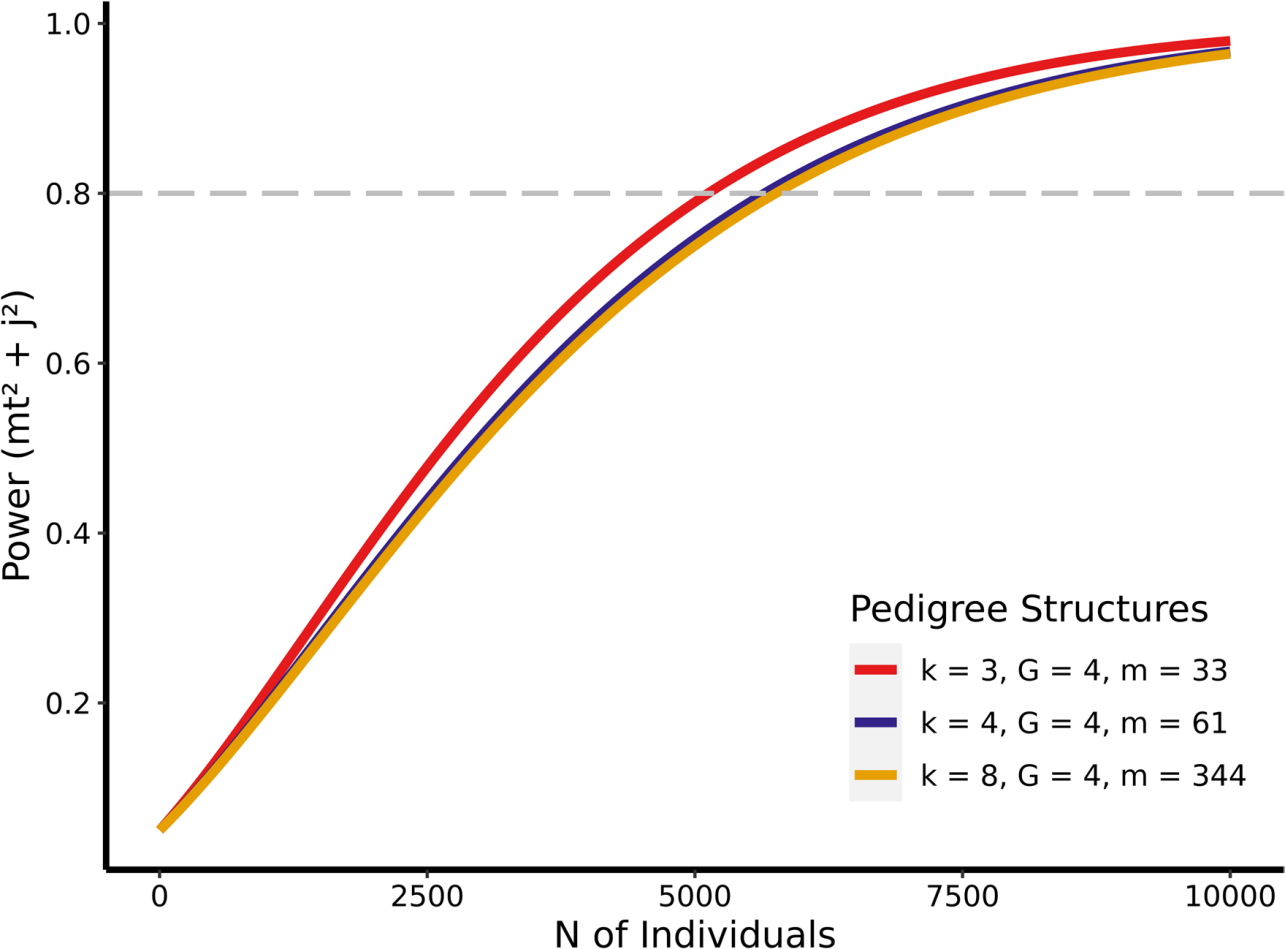
Estimation power for $Mt^2$ and $J^2$ as a function of total sample sizes and number of offspring per mate in simulated pedigrees. *k* number of offspring per mate, *G* number of generations, *m* pedigree size. Effect sizes of variance components: $a^2 = 0.40$, $i^2 = 0$, $mt^2 =.05$, $c_F^2 =.1$, $c_E^2 =.1$, and $j^2 =.01$

We find that the average number of offspring per mate (*k*) has a lower influence on the estimation of mtDNA effects, in comparison with the impact of number of generations (*G*). Our findings reveal that a higher number of offspring per couple leads to slightly decreased power to detect mtDNA effects, as demonstrated in Fig. 5. In particular, for medium mtDNA effects ($mt^2$ =.05, $j^2$ =.01), a sample size of 5755 individuals from pedigrees with an average of eight offspring per mate (*k* = 8) is necessary to achieve a power of.8 when using a four-generation deep pedigree. However, when each mate has three offspring on average (*k* = 3), only 5124 individuals are needed to reach a power of.8, resulting in greater power than with a larger nuclear family.

### Model Misspecification

#### Mutation

**Fig. 6:**
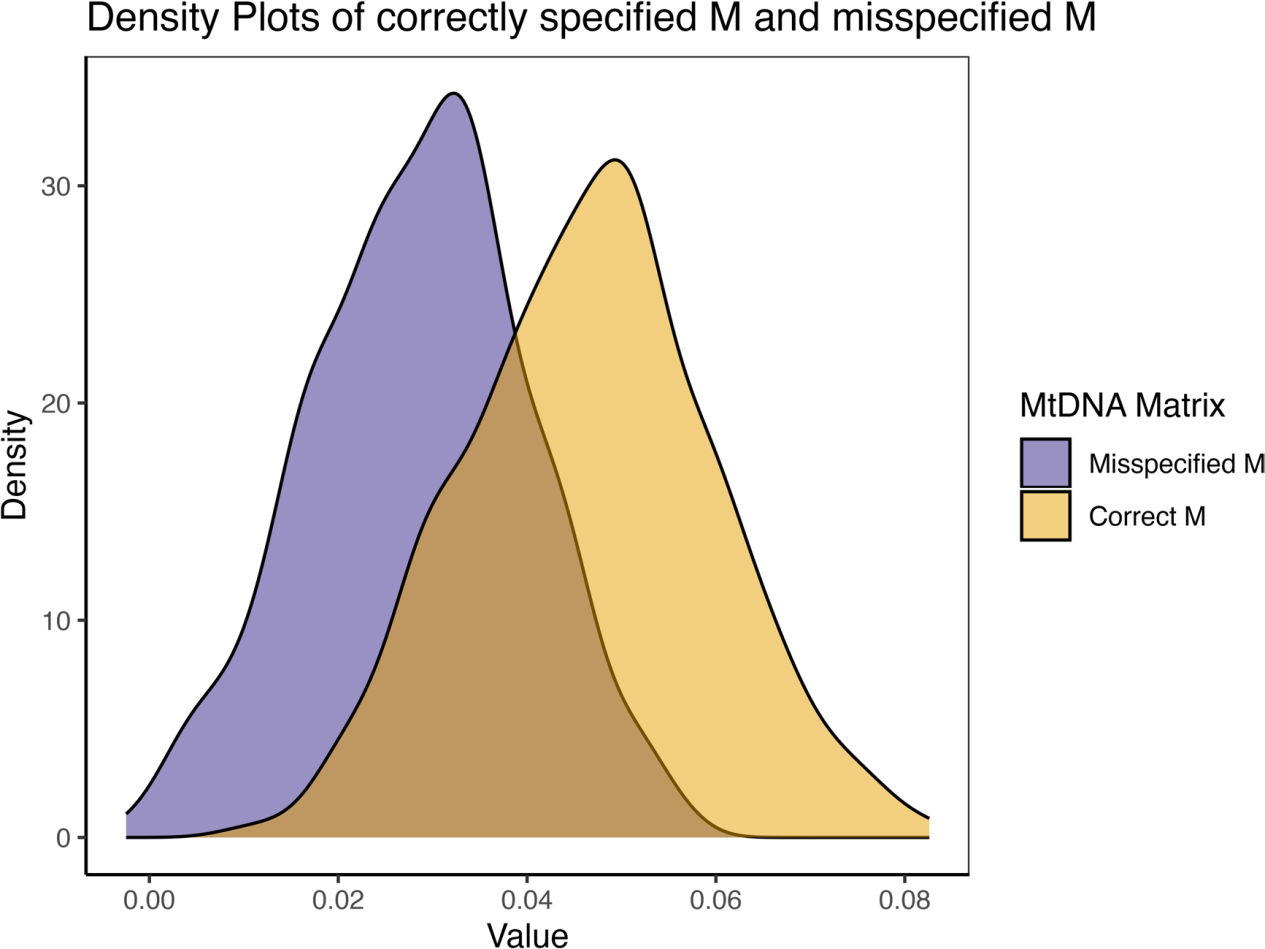
Density plot of 500 $mt^2$ estimates. The purple shade is the biased estimation: fitting the model with relatedness matrices which includes one undetected mtDNA mutation in one second-generation female member from the pedigree. The yellow shade is the unbiased estimation: fitting the model with correct relatedness matrices

In our simulation, we introduced a single nucleotide mutation in the mtDNA at the time of gamete formation for a single female individual in the second generation. When this mutation is not correctly specified, it leads to a cascade of misspecifications in her offspring. Across randomly simulated pedigrees, we found that $mt^2$ is underestimated by 38.86%. This is demonstrated in Fig. 6, where the mean $mt^2$ estimates from 500 simulations decreased from.0466 to.0285 when the simulated population level was.05. Conversely, $j^2$ parameters are drastically overestimated, deviating from.0175 to.0563.

#### Missing Links

**Fig. 7:**
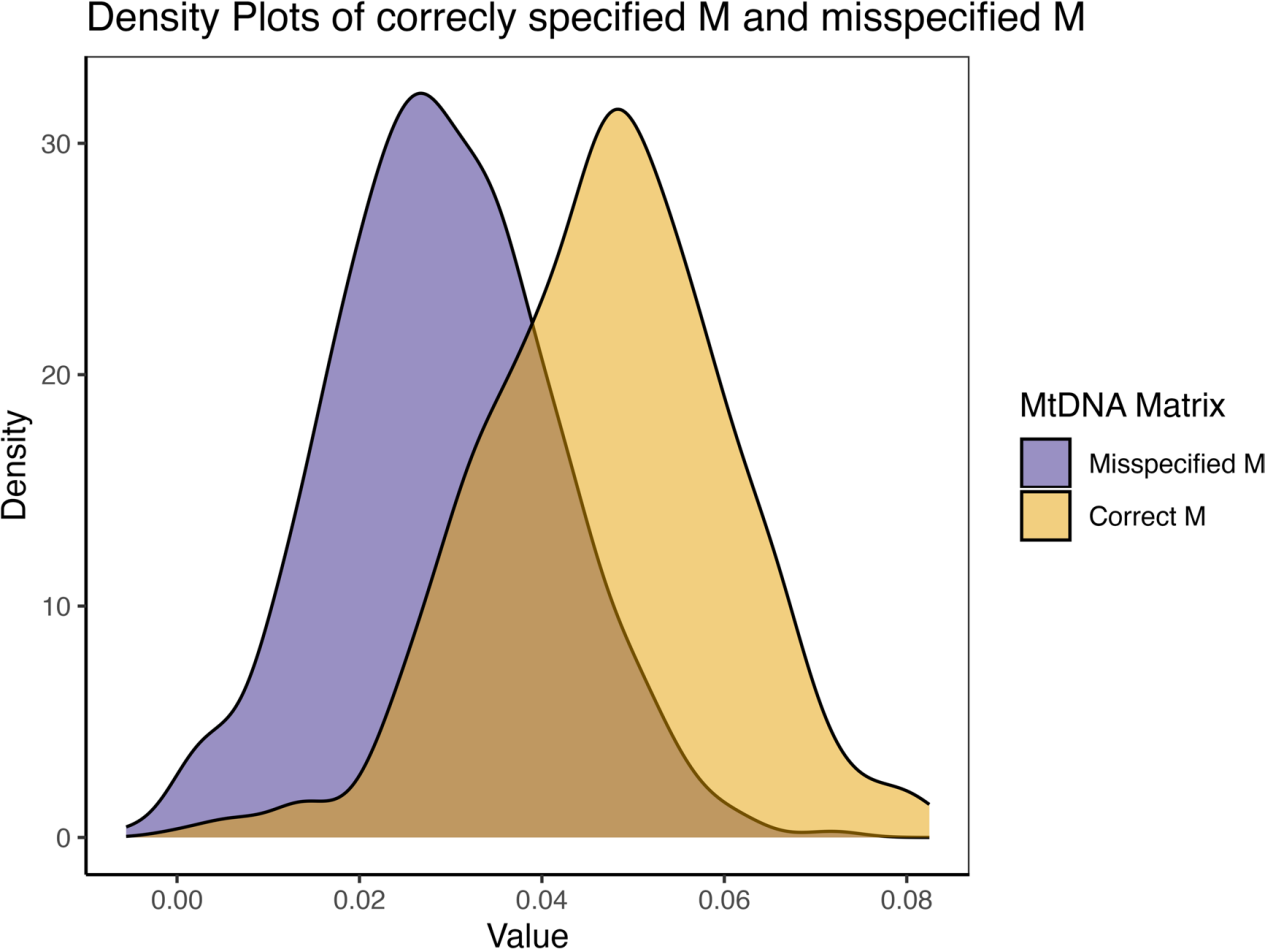
Density plot of 500 $mt^2$ estimates contrasting biased and unbiased estimations. The biased estimation (purple shade) involves fitting the model with relatedness matrices that include an undetected mtDNA mutation in a second-generation female member of the pedigree. The unbiased estimation (yellow shade) involves fitting the model with correct relatedness matrices

We find that $mt^2$ is underestimated by 38.65% if we do not correctly specify the parent-offspring relationship for a female offspring from the second generation. Demonstrated in Figure 7, the mean $mt^2$ estimates from the 500 simulations reduced from.0472 to.0289, when the simulated population level is.05. Similar to the unidentified mutation case, $j^2$ parameters are overestimated, increasing from.0186 to.0606.

#### False Positives Under the Null Hypothesis

**Fig. 8:**
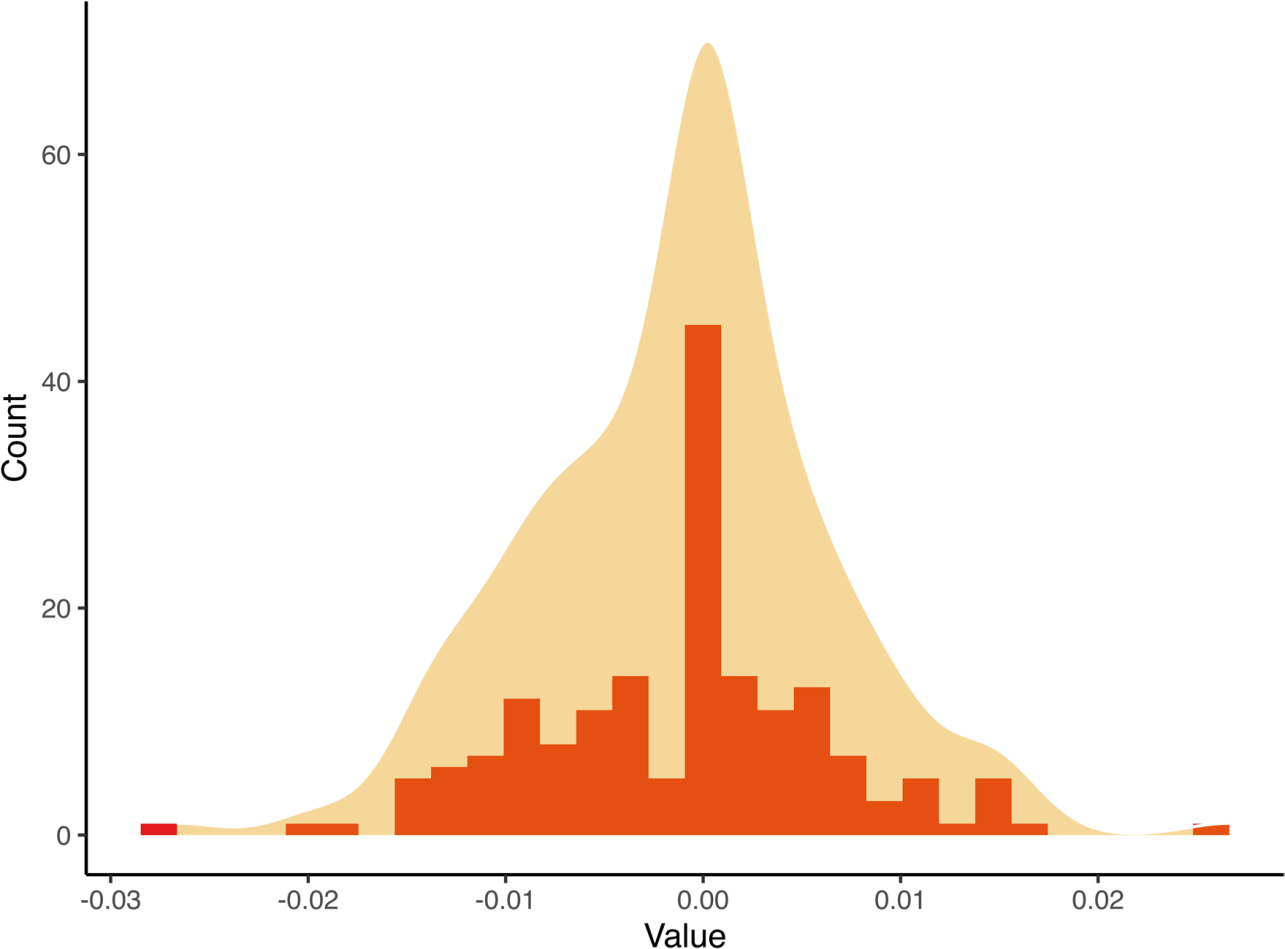
Histogram and density plot showing the distribution of $mt^2$ estimates across 500 simulations under the null hypothesis, centered around the expected value of zero

To evaluate our extended pedigree model’s performance under the null hypothesis-where no mtDNA effects ($mt^2$ and $j^2$) are expected - we simulated data under the null model ($mt^2 = 0$; $j^2 = 0$) and fit that data to the full mtDNA model. As shown in Figure 8, the estimates for $mt^2$ and $j^2$ were unbiased and did not result in excessive false positives. Across the 200 simulated models, $mt^2$ have a mean of -.001 and a standard deviation of.0006. $j^2$ have a mean of.005 and a standard error of.0019. Further, 80.80% of the $mt^2$ estimates fell within a 0.01 deviation from 0 (the true population parameter), and all were within a 0.03 deviation, while 44.07% of $j^2$ estimates fell within the same 0.01 range. For any traits with a true $mt^2$ estimate greater than.03, the p value for the test will be 0 when the sample size is not less than 20,000.

Overall, the results indicate that our mtDNA estimates are reasonably robust to assumption violations. In cases of extreme misspecification, the inability to detect missing maternal linkage and failure to account for mtDNA mutation led to a slightly underestimated variance of mtDNA at the population level. Furthermore, when fitting the model with data simulated under the null model, a modest false positive estimate in $mt^2$ estimates was introduced.

## Discussion

The current study investigated the statistical power and estimation bias when using an extended pedigree model to estimate mitochondrial DNA (mtDNA) effects. Our findings suggest that a sample size of approximately 5,000 is generally adequate to detect an mtDNA effect size of 5%. Our findings indicate that greater additive genetic variance the statistical power for detecting mtDNA effects, whereas greater nuclear environmental variance diminishes this power. We found that larger additive genetic variance will increase power to detect mtDNA effects, but larger nuclear environmental variance will decrease it. Additionally, our examination of estimation bias underscores the robustness of the extended pedigree model in estimating mtDNA effects. Specifically, failure to account for missing kinships in the pedigree and possible mtDNA mutation both lead to slight underestimates of mtDNA variance. Overall, the false positive rate is modest for mtDNA estimation from the extended pedigree model. Collectively, these results support the extended pedigree model’s efficacy and validity for investigating the influence of mtDNA on human traits.

The power to detect mtDNA increased with larger effect sizes, as demonstrated by our simulations with mtDNA effect sizes ranging from large ($mt^2 =.10$) to small ($mt^2 =.005$). This trend aligns with previous power analyses for additive genetic effect estimation and common environmental effects estimated through classical twin and other family designs (Posthuma and Boomsma 2000; Sham et al. 2020; Verhulst 2017; Visscher 2004; Visscher et al. 2008). Moreover, in the broader context of structural equation modeling (SEM), effect size is consistently identified as one of the most critical factors affecting power (Curran et al. 2002; MacCallum et al. 1996; Wang and Rhemtulla 2021). Besides an increase in the effect size of the targeted effects themselves, we found that increase in additive genetic variance leads to higher power for the estimation of the mtDNA effects, paralleling from ACE or ADE models which show that greater variance attributed to common environmental effects ($c^2$) improves power for $a^2$ estimates (Posthuma and Boomsma 2000; Verhulst 2017). Interestingly, an increased magnitude of nuclear family effect variance ($c_F^2$) reduces the statistical power for estimating mtDNA effects. This reduction occurs primarily because the relatedness matrix for nuclear family effects often exhibits considerable similarity to the mtDNA relatedness matrix, particularly within smaller pedigrees. Consequently, when the $c_F^2$ component is substantial, it becomes more challenging to accurately distinguish the (often smaller) variance attributable to mtDNA from that due to pervasive nuclear family effects. This difficulty arises because their respective contributions to phenotypic variance can be partially confounded by similarities in the underlying patterns of familial resemblance they induce. Therefore, larger samples and greater variety in family structure are generally required to effectively disentangle these two sources of variance and robustly estimate mtDNA contributions.

Our study defined pedigree width as the number of offspring per mate (*k*) and depth as the total number of generations (*G*), which are the two main factors influencing the shape of family trees. We found that a larger *G* leads to a greater power contribution per individual, but a larger *k* decreased it. In other words, data with more far-reached ancestries will grant researchers advantages in detecting mtDNA effects while a contemporary and more fecund population may be less ideal for such analysis, given the same amount of individuals in the data. The advantage of deeper pedigrees lies in their ability to more clearly distinguish between the effects of nuclear and mitochondrial effects. Conversely, wider pedigrees often yield redundant information to parse mtDNA effects from nuclear DNA. To understand the separation of $a^2$ from $mt^2$, we need to recognize that all members within the same generation either share a 0.5 additive genetic relatedness as siblings or a 0.125 as cousins. In contrast, between-generation kinship varies more in additive genetic relatedness. Therefore, "wide" pedigrees have more kinship links with 0.5 and 0.125 expected additive genetic relatedness, while "deep" pedigrees have links with expected values less than 0.125. At the same time, both the "wide" and "deep" pedigrees have roughly the same proportion of kinship that share the same mtDNA. Consequently, the effects of mtDNA will be easier to manifest in the "deep" pedigree because the influence on similarity between a pair of members, stemming from mtDNA, can be more easily separated from the similarity originating from nuclear DNA. This is due to the smaller average expected nuclear genetic relatedness values in the "deep" pedigrees. In other words, collecting data with more embedded generations or expanding existing datasets to include information from earlier and later generations can be a viable approach to increasing power.

The current model assumes that kinship records are entirely accurate and that mtDNA mutations are absent. Yet, these conditions are rarely met in practice. Our bias analysis indicated that mtDNA effects are underestimated when relatedness matrices are misspecified. Given that the extended pedigree model is intended to pioneer research on mtDNA effects on specific traits, underestimating these effects is preferable to committing a type-I error. Consequently, researchers can be confident when they uncover significant mtDNA variance, even in samples that have imperfect kinship records. Moreover, our scenarios of missing kinship and ignored mutations involve extreme misspecifications of relatedness matrices, scenarios unlikely in real-world settings. For instance, our analysis of bias from missing kinship excluded an entire maternal branch from the second generation across all simulated pedigrees. In actual data, such extensive omissions do not occur in all families, nor are all missed kinship links concentrated on the maternal line. Furthermore, while unidentified kinship may exist across generations, omissions outside the second generation should introduce less bias on the construction of relatedness matrices. Even under this extreme scenario, the estimates of $mt^2$ diminished by approximately 40% when a single critical branch was removed from the pedigree. In practice, the impact of such misspecification is much less profound in empirical settings. The same discrepancy exists between the simulation of unidentified mtDNA mutation and the occurrence of these mutations in real data. However, we find that non-identified mtDNA mutation is linked with a 35% decrease of the $mt^2$ estimates. Furthermore, we find that both unidentified kinship and unidentified mtDNA mutation leads to a strong overestimation of $j^2$ estimates. The similarity between the related matrices of $a^2$ and $j^2$ are the primary force driving the overestimation. Based on these results, we recommend users of the current model to be more cautious about interpreting the results when the $j^2$ estimate surpasses the $mt^2$ estimate in the model fitting results, as the pattern could be a strong indication of incorrectly specified relatedness matrices. Recent advancements in low-cost sequencing technology can aid in identifying unrecorded kinship and correcting any inaccuracies in recorded kinship. However, currently, there are very few datasets that include both extensive pedigrees and genomic information across generations, with MoBa as an exception (Magnus et al. 2016).

The robustness of our model is further evidenced by its performance under the null model, when no mtDNA effects are present. Specifically, only a small proportion of mtDNA estimates$-$19.20%-deviated from zero by more than 0.01, underscoring the model’s capacity to distinguish true mtDNA variance from noise. Therefore, it is very unlikely to misattribute other sources of variance to mtDNA, if the mtDNA effects account for more than 2% of the total phenotypic variance. Datasets that have records of extended pedigrees typically encompass hundreds of thousands of individuals. For example, the Utah Population Database (UPB Smith et al. 2022; Bean et al. 1978) has over 11 million people nested within pedigrees, crowd-sourced pedigrees hosted on Geni.com contain over 86 million people (including a single pedigree with 13 million people, Kaplanis et al. 2018) in publicly available pedigrees, and the Norwegian Mother, Father and Child Cohort Study (MoBa Magnus et al. 2016) has at least 95,000 families, sizes that are more than sufficient to detect mtDNA effects. The low false positive rate can ease researchers and funding agencies’ anxiety about spending efforts and funding on a spurious research direction. On the other side of the coin, the usage of current models will provide reliable insights on revealing the role of mtDNA in various phenotypes and effectively guide the future direction.

### Limitations and Future Directions

One limitation of the power analysis is that we only investigated continuous traits. Abundant evidence in family designs has suggested dichotomous traits will lead to a significant drop in bias (M.C. Neale et al. 1994). We also anticipate the power for mtDNA estimation will decrease when applying the model to a binary outcome. Based on our results from a continuous outcome, large pedigree datasets with more than 20,000 individuals should be sufficient to detect a mtDNA effect of no less than 1%. However, an accurate power curve needs a similar simulation design to the current study so that it can be precisely derived.

One other notable limitation of the current study is the use of fixed-generation pedigrees for each condition in the simulations. Specifically, each group consisting of 20,000 simulated individuals has the same number of generations, though the number of offspring per mate varies among different mates following a Poisson distribution. The current approach offers advantages in power calculation because we can obtain an accurate unit non-centrality parameter for all sample sizes under the same conditions (Dominicus et al. 2006; Satorra and Saris 1985). However, real-life data more closely resembles a combination of pedigrees with varying depth (Mawass and Milot 2025). The impact of pedigree structure will qualitatively hold true, but the sample sizes to reach a certain threshold of power will be different.

We did not craft simulations for all possible violations of assumptions, focusing instead on those we deemed most critical for this model-unidentified kinship and mtDNA mutations. We did not include other potential misspecifications that also bias the relatedness matrices. These potential misspecifications include treating monozygotic twins and half-siblings as full siblings, as well as structural issues like endogamy (marriage within a specific community), pedigree collapse (where ancestors appear multiple times in a pedigree), and genealogical error. These scenarios are less common and generally result in only modest alterations to the relatedness matrices, thereby introducing less substantial bias compared to the primary issues we addressed. For researchers concerned that these less common scenarios might substantially affect their particular sample results, our (BGmisc Garrison et al. 2024), provides tools designed to analyze such biases, including specialized functionality for twinning and pedigree collapse. (See Hunter et al. 2024, for additional discussion on these tools.)

Another limitation is that the current model has not considered the impact of assortative mating on the parameter estimation. We acknowledge that assortative mating, while not explicitly modeled in the current study, can potentially introduce bias in parameter estimates by misattributing the variance it induces. However, we anticipate that estimates of mtDNA variance may be relatively robust to this bias. This is because the covariance patterns arising from assortative mating are likely to align more closely with those captured by shared environmental components (e.g., nuclear family, $\mathbf {C_F}$, or extended family, $\mathbf {C_E}$) than with the distinct inheritance pattern of mtDNA (represented by $\textbf{M}$); furthermore, the strength of assortative mating is known to be trait-dependent. Comprehensively addressing assortative mating, whether through statistical adjustment for primary assorting variables or through direct modeling of its effects, presents significant methodological challenges, including potential inaccuracies, substantial computational demands for complex pedigrees, and sensitivity to model misspecification. Therefore, while a full quantitative investigation and integration of assortative mating effects are beyond the current scope, they represent an important avenue for future research and model refinement.

The model offers researchers a robust tool to analyze the contribution of mtDNA to human complex traits. A key strength of the model lies in its ability to separate mtDNA effects from other maternal influences. First, some males share the same mtDNA as their female siblings, which helps isolate mtDNA effects from sex- and gender-based effects. Second, maternal environmental effects often weaken with increasing familial distance within a pedigree. For instance, while maternal antecedents may influence certain traits, the impact diminishes with greater degrees of relatedness, such as the weaker influence of female grandparents compared to female parents. In contrast, mtDNA effects remain consistent throughout the maternal lineage, from grandparents to offspring. Although we did not explicitly model the effects of female parents, we believe these effects are less significant compared to other environmental factors. Thus, mtDNA effects are fundamentally distinct from environmental influences and are not confounded by them, especially with deep pedigrees. Future studies should explore potential biases in mtDNA estimation that could result from omitting other maternally related effects.

#### Future Directions

There are numerous potential future directions for modeling the effects of mtDNA in large pedigrees. Our team has applied this model to studies on longevity and Alzheimer’s disease, and we observed some positive indications of mtDNA effects on both traits (Burt 2023; Burt et al. 2024). In addition to empirical applications, several modifications can be made to enhance the current model. First, similar to other covariance structure models used in behavioral genetics, this model could be extended for multivariate analysis, which would allow for the estimation of shared mtDNA effects between two or more traits.

Second, mtDNA effects can be incorporated into extended family biometrical models as an additional source of phenotypic variance. This approach is particularly valuable in designs that include maternal and paternal relatives across a range of genetic distances, facilitating the isolation of matrilineal inheritance patterns. Furthermore, the model allows for the inclusion or control of other relevant factors that might influence phenotypes:

1. To differentiate specific environmental or non-mtDNA genetic influences transmitted through the maternal line, researchers could model the impact of maternal grandparents by assessing defined grandparent-offspring interactions, after accounting for potential mtDNA-related confounding.
2. The models can be extended to estimate variance components associated with other maternal environmental factors, such as the mother’s working status (e.g., stay-at-home versus working), by incorporating appropriate indicators.
3. Influences of family structure itself (e.g., sibship size, parental age, or birth order) on phenotypes may also be explicitly modeled within this framework, thereby helping to disentangle the various sources of familial resemblance and more accurately estimate other genetic and environmental contributions, including those from mtDNA.

Third, a key consideration for our findings, particularly those derived from power analyses of deep pedigrees, is the potential influence of cohort effects. Our current models demonstrate statistical advantages under assumed stable environmental conditions, but we acknowledge that over the multi-generational timespans inherent in such pedigrees, significant societal changes (e.g., in educational access, healthcare, or nutrition) can introduce cohort-specific variations in phenotypes. This could inflate total phenotypic variance and potentially alter the estimated magnitude of genetic contributions, including those from mtDNA. Nevertheless, the distinct binary inheritance pattern of mtDNA-contrasting with the more gradual and pervasive nature of societal shifts-suggests that its effects should remain distinguishable. For some traits, applying within-generation standardization (e.g., using z-scores) can help maintain measurement validity across cohorts, although traits whose fundamental definitions may evolve over time warrant more cautious interpretation.

Addressing cohort effects more directly within the modeling framework presents both opportunities and challenges. Although approaches such as time-point stratification are conceivable for traits with clear historical transitions, this often comes at the cost of significantly reduced statistical power, potentially obscuring smaller genetic effects. A more robust long-term solution would involve explicitly incorporating cohort effects as a distinct variance component within extended pedigree models. This could potentially be achieved by extending current specifications, perhaps drawing from multi-group structural equation modeling or Bayesian frameworks, to include cohort-specific parameters (e.g., a cohort-specific variance component). Developing and validating such sophisticated models tailored for extended pedigree designs represents a significant but important avenue for future research.

Fourth, with the advent of genotyped family databases, it is now highly feasible to model the influence of specific mtDNA haplogroups alongside additive genetic and environmental effects, which could provide deeper insights into the biological impacts of mtDNA on complex human traits. Lastly, extended pedigree data typically encompass individuals from multiple generations. Therefore, future modifications should incorporate cohort effects as an additional source of variance.

## Conclusion

In the current study, we proposed and evaluated an extended pedigree model to quantify the influence of mitchochondrial DNA (mtDNA) on human behavior, controlling for influences from other genetic and environmental sources. The statistical power and bias analysis of the mtDNA effect estimate suggests that the model is sufficiently robust as a pioneering qualitative and quantitative exploration of the potential impact of mtDNA. The model is a powerful enough tool to detect medium to small mtDNA effects (5% of total variance) with a medium sample size (around 5,000). Nevertheless, we found that pedigree structures have a moderate influence on the statistical power of mtDNA effect estimation. Specifically, pedigrees with more generations typically increase power, while pedigrees with more offspring per mate typically decrease power, given the same total number of individuals involved. In terms of estimation bias, not having accurate kinship information and not accounting for mtDNA mutation underestimates the mtDNA variance by 35% to 40% under extremely misspecified scenarios. In addition, the mtDNA variance estimate has a small false positive rate. These findings substantiate the model’s value in examining the role of mtDNA in human behavior and open up numerous opportunities for designing novel biometrical models and investigating new research questions that can possibly be answered with extended pedigrees in behavior genetics.

## Data Availability

All simulation codes for the analyses can be found on https://github.com/Xuanyu-Lyu/mtDNA. Due to the size of the simulated data, the data itself is not provided in the Github repository above.

## Footnotes

1 Although these parameters are in the formats of squared coefficients, we do not impose a lower bound of 0 when estimating them in OpenMx.

2 In this paper, we define pedigree as any maximal set of individuals among whom each pair is joined by a sequence of one or more parent–offspring or mate relationships.

3 In this paper, we use ’sex’ to describe biological differences determined by chromosomes (e.g., XX, XY, or other variations). These genotypic differences are distinct from the phenotypic expression of ’gender’. While ’gender’ encompasses the broader, richer, personal, deeply-held sense of being male, female, a blend of both, neither, or another gender entirely, our simulation’s focus is strictly on building pedigrees.

4 Mutation rate estimates vary depending on how they’re derived (Parsons et al. 1997). For a recent commentary, see (Schaack et al. 2019). Empirically derived estimates can potentially be as high as once every 30 (Connell et al. 2022).

## References

[CR1] Anderson S, Bankier AT, Barrell BG, de Bruijn MHL, Coulson AR, Drouin J, Young IG (1981) Sequence and organization of the human mitochondrial genome. Nature 290(5806):457–465. 10.1038/290457a0 7219534 10.1038/290457a0

[CR2] Ayala-Peña S (2013) Role of oxidative DNA damage in mitochondrial dysfunction and Huntington’s disease pathogenesis. Free Radical Biol Med 62:102–110. 10.1016/j.freeradbiomed.2013.04.017 23602907 10.1016/j.freeradbiomed.2013.04.017 PMC3722255

[CR3] Bean LL, May DL, Skolnick M (1978) The Mormon historical demography project. Historical Methods: J Quant Interdiscip History 11:45–53. 10.1080/01615440.1978.9955216 10.1080/01615440.1978.9955216 11614600

[CR4] Borger MJ, Komdeur J, Richardson DS, Weissing FJ (2023) The estimation of reproductive values from pedigrees. Behav Ecol 34(5):850–861. 10.1093/beheco/arad049 37744170 10.1093/beheco/arad049 PMC10516676

[CR5] Burt SA (2023) Mom genes: leveraging maternal lineage to estimate the contributions of mitochondrial dna. Behav Genet 233:10013

[CR6] Burt SA, Garrison SM, Lyu X, Good R, Carroll SL, Coon H, Hunter MD (2024) Exploring the influence of mitochondrial dna on longevity using the utah population database. Preprint, Preprint

[CR7] Castellani CA, Longchamps RJ, Sun J, Guallar E, Arking DE (2020) Thinking outside the nucleus: mitochondrial dna copy number in health and disease. Mitochondrion 53:214–223. 10.1016/j.mito.2020.06.004 32544465 10.1016/j.mito.2020.06.004 PMC7375936

[CR8] Cha M-Y, Han S-H, Son SM, Hong H-S, Choi Y-J, Byun J, Mook-Jung I (2012) Mitochondria-specific accumulation of amyloid [CDATA[\beta ]] induces mitochondrial dysfunction leading to apoptotic cell death. PLoS ONE 7(4):e34929. 10.1371/journal.pone.0034929 22514691 10.1371/journal.pone.0034929 PMC3325919

[CR9] Cha M-Y, Kim DK, Mook-Jung I (2015) The role of mitochondrial DNA mutation on neurodegenerative diseases. Exp Mol Med 47(3):e150–e150. 10.1038/emm.2014.122 25766619 10.1038/emm.2014.122 PMC4351410

[CR10] Chen W-M, Abecasis GR (2006) Estimating the power of variance component linkage analysis in large pedigrees. Genet Epidemiol 30(6):471–484. 10.1002/gepi.20160 16685720 10.1002/gepi.20160

[CR11] Chong M, Mohammadi-Shemirani P, Perrot N, Nelson W, Morton R, Narula S, Paré G (2022) Gwas and exwas of blood mitochondrial dna copy number identifies 71 loci and highlights a potential causal role in dementia. Elife 11:e70382. 10.7554/eLife.70382 35023831 10.7554/eLife.70382 PMC8865845

[CR12] Cohen J (1988) Statistical power analysis for the behavioral sciences, 2nd edn. Routledge, New York

[CR13] Cohen J (1992) Statistical power analysis. Curr Dir Psychol Sci 1(3):98–101. 10.1111/1467-8721.ep10768783

[CR14] Connell JR, Benton MC, Lea RA, Sutherland HG, Chaseling J, Haupt LM, Griffiths LR (2022) Pedigree derived mutation rate across the entire mitochondrial genome of the norfolk island population. Sci Rep 12(1):6827. 10.1038/s41598-022-10530-3 35473946 10.1038/s41598-022-10530-3 PMC9042960

[CR15] Curran PJ, Bollen KA, Paxton P, Kirby J, Chen F (2002) The noncentral chi-square distribution in misspecified structural equation models: finite sample results from a Monte Carlo simulation. Multivar Behav Res 37(1):1–36. 10.1207/S15327906MBR3701_01 10.1207/S15327906MBR3701_01 26824167

[CR16] DiMauro S, Schon EA (2001) Mitochondrial dna mutations in human disease. Am J Med Genet 106(1):18–26. 10.1002/ajmg.1392 11579421 10.1002/ajmg.1392

[CR17] Dominicus A, Skrondal A, Gjessing HK, Pedersen NL, Palmgren J (2006) Likelihood ratio tests in behavioral genetics: problems and solutions. Behav Genet 36(2):331–340. 10.1007/s10519-005-9034-7 16474914 10.1007/s10519-005-9034-7

[CR18] Felson J (2014) What can we learn from twin studies? A comprehensive evaluation of the equal environments assumption. Soc Sci Res 43:184–199. 10.1016/j.ssresearch.2013.10.004 24267761 10.1016/j.ssresearch.2013.10.004

[CR19] Ferreira T, Rodriguez S (2024) Mitochondrial dna: inherent complexities relevant to genetic analyses. Genes 15(5):617. 10.3390/genes15050617 38790246 10.3390/genes15050617 PMC11121663

[CR20] Garrison SM, Hunter MD, Lyu X, Good RN, https://www.jdtrat.com/), JDTu, Burt SA (2025) BGmisc: an r package for extended behavior genetics analysis. Retrieved 2025-06-12 from https://cran.r-project.org/web/packages/BGmisc/index.html 10.21105/joss.06203 PMC12308509 40741040

[CR21] Garrison SM (2025) ggpedigree: visualizing pedigrees with ’ggplot2’ and ’plotly’. Retrieve 2025 from https://cran.r-project.org/web/packages/ggpedigree/index.html 10.21105/joss.09434 PMC13193231 42181847

[CR22] Garrison SM, O’Keefe P, Hunter M, Beasley W, Bard D, Rodgers J (2018) AC’RE model: estimating rearing effects without twins raised apart. Behav Genet 48:472–472

[CR23] Garrison SM, Hunter MD, Lyu X, Trattner JD, Burt SA (2024) Bgmisc: an r package for extended behavior genetics analysis. J Open Source Softw 9(94):6203. 10.21105/joss.06203 40741040 10.21105/joss.06203 PMC12308509

[CR24] Giles RE, Blanc H, Cann HM, Wallace DC (1980) Maternal inheritance of human mitochondrial DNA. Proc Natl Acad Sci USA 77(11):6715–6719. 10.1073/pnas.77.11.6715 6256757 10.1073/pnas.77.11.6715 PMC350359

[CR25] Gruschus JM, Morris DL, Tjandra N (2023) Evidence of natural selection in the mitochondrial-derived peptides humanin and shlp6. Sci Rep 13(1):14110. 10.1038/s41598-023-41053-0 37644144 10.1038/s41598-023-41053-0 PMC10465549

[CR26] Guyatt AL, Brennan RR, Burrows K, Guthrie PAI, Ascione R, Ring SM, Rodriguez S (2019) A genome-wide association study of mitochondrial dna copy number in two population-based cohorts. Hum Genomics 13(1):6. 10.1186/s40246-018-0190-2 30704525 10.1186/s40246-018-0190-2 PMC6357493

[CR27] Hunter MD, Garrison SM, Lyu X, Good R, Carroll SL, Burt SA (2024) Tracing the right path: determination of large pedigree segmentation and relatedness. Preprint submitted to APA Journal, Preprint 10.1007/s10519-026-10259-z PMC13421354 42225929

[CR28] Hunter MD, Garrison SM, Burt SA, Rodgers JL (2021) The analytic identification of variance component models common to behavior genetics. Behav Genet 51(4):425–437. 10.1007/s10519-021-10055-x 34089112 10.1007/s10519-021-10055-x PMC8394168

[CR29] Israelson A, Arbel N, Da Cruz S, Ilieva H, Yamanaka K, Shoshan-Barmatz V, Cleveland DW (2010) Misfolded mutant SOD1 directly inhibits VDAC1 conductance in a mouse model of inherited ALS. Neuron 67(4):575–587. 10.1016/j.neuron.2010.07.019 20797535 10.1016/j.neuron.2010.07.019 PMC2941987

[CR30] Jin H, Kanthasamy A, Ghosh A, Anantharam V, Kalyanaraman B, Kanthasamy AG (2014) Mitochondria-targeted antioxidants for treatment of Parkinson’s disease: preclinical and clinical outcomes. Biochimica et Biophysica Acta (BBA) - Mol Basis Dis 1842(8):1282–1294. 10.1016/j.bbadis.2013.09.007 10.1016/j.bbadis.2013.09.007 PMC3961561 24060637

[CR31] Jobst L, Bader M, Moshagen M (2021) A tutorial on assessing statistical power and determining sample size for structural equation models. Psychol Methods. 10.1037/met0000423 34672644 10.1037/met0000423

[CR32] Kaplanis J, Gordon A, Shor T, Weissbrod O, Geiger D, Wahl ME, Erlich Y (2018) Quantitative analysis of population-scale family trees with millions of relatives. Science 360:171–175. 10.1126/science.aam9309 29496957 10.1126/science.aam9309 PMC6593158

[CR33] Keller MC, Medland SE, Duncan LE (2010) Are extended twin family designs worth the trouble? A comparison of the bias, precision, and accuracy of parameters estimated in four twin family models. Behav Genet 40(3):377–393. 10.1007/s10519-009-9320-x 20013306 10.1007/s10519-009-9320-x PMC3228846

[CR34] Lima T, Li TY, Mottis A, Auwerx J (2022) Pleiotropic effects of mitochondria in aging. Nat Aging 2(3):199–213. 10.1038/s43587-022-00191-2 37118378 10.1038/s43587-022-00191-2

[CR35] Lin MT, Beal MF (2006) Mitochondrial dysfunction and oxidative stress in neurodegenerative diseases. Nature 443(7113):787–795. 10.1038/nature05292 17051205 10.1038/nature05292

[CR36] Lyu X, Garrison SM (2023) Effects of genetic relatedness of kin pairs on univariate ace model performance. Twin Res Hum Genet 26:257–268. 10.1017/thg.2023.40 37799059 10.1017/thg.2023.40 PMC11421410

[CR37] MacCallum RC, Browne MW, Sugawara HM (1996) Power analysis and determination of sample size for covariance structure modeling. Psychol Methods 1(2):130–149. 10.1037/1082-989X.1.2.130

[CR38] Magnus P, Birke C, Vejrup K, Haugan A, Alsaker ER, Daltveit AK, Stoltenberg C (2016) Cohort profile update: the norwegian mother and child cohort study (moba). Int J Epidemiol 45:382–388. 10.1093/ije/dyw029 27063603 10.1093/ije/dyw029

[CR39] Marande W, Burger G (2007) Mitochondrial DNA as a genomic jigsaw puzzle. Science 318(5849):415–415. 10.1126/science.1148033 17947575 10.1126/science.1148033

[CR40] Martin NG, Eaves LJ, Kearsey MJ, Davies P (1978) The power of the classical twin study. Heredity 40(1):97–116. 10.1038/hdy.1978.10 272366 10.1038/hdy.1978.10

[CR41] Mawass W, Milot E (2025) Assessing the impact of pedigree attributes on the validity of quantitative genetic parameter estimates. J Evol Biol 38(4):439–456. 10.1093/jeb/voaf010 39903138 10.1093/jeb/voaf010

[CR42] Moraes CT, Srivastava S, Kirkinezos I, Oca-Cossio J, vanWaveren C, Woischnick M, Diaz F (2002) Mitochondrial dna structure and function. Int Rev Neurobiol 53:3–23 12512335 10.1016/s0074-7742(02)53002-6

[CR43] Murakami T, Nagai M, Miyazaki K, Morimoto N, Ohta Y, Kurata T, Abe K (2007) Early decrease of mitochondrial DNA repair enzymes in spinal motor neurons of presymptomatic transgenic mice carrying a mutant SOD1 gene. Brain Res 1150:182–189. 10.1016/j.brainres.2007.02.057 17434152 10.1016/j.brainres.2007.02.057

[CR44] Neale MC, Eaves LJ, Kendler KS (1994) The power of the classical twin study to resolve variation in threshold traits. Behav Genet 24(3):239–258. 10.1007/BF01067191 7945154 10.1007/BF01067191

[CR45] Neale M, Cardon LR (2003) Methodology for genetic studies of twins and families. Springer

[CR46] Nicholls TJ, Minczuk M (2014) In d-loop: 40 years of mitochondrial 7s dna. Exp Gerontol 56:175–181. 10.1016/j.exger.2014.03.027 24709344 10.1016/j.exger.2014.03.027

[CR47] Oliveira JMA, Lightowlers RN (2010) Could successful (mitochondrial) networking help prevent Huntington’s disease? EMBO Mol Med 2(12):487–489. 10.1002/emmm.201000104 21117121 10.1002/emmm.201000104 PMC3377349

[CR48] Parsons TJ, Muniec DS, Sullivan K, Woodyatt N, Alliston-Greiner R, Wilson MR, Holland MM (1997) A high observed substitution rate in the human mitochondrial DNA control region. Nat Genet 15(4):363–368. 10.1038/ng0497-363 9090380 10.1038/ng0497-363

[CR49] Posthuma D, Boomsma DI (2000) A note on the statistical power in extended twin designs. Behav Genet 30(2):147–158. 10.1023/A:1001959306025 10979605 10.1023/a:1001959306025

[CR50] Rao DC, Vogler GP, McGue M, Russell JM (1987) Maximum-likelihood estimation of familial correlations from multivariate quantitative data on pedigrees: a general method and examples. Am J Hum Genet 41(6):1104–1116 3687943 PMC1684353

[CR51] Reddy PH (2009) Amyloid beta, mitochondrial structural and functional dynamics in Alzheimer’s disease. Exp Neurol 218(2):286–292. 10.1016/j.expneurol.2009.03.042 19358844 10.1016/j.expneurol.2009.03.042 PMC2710427

[CR52] Rodgers JL, Garrison SM, O’Keefe P, Bard DE, Hunter MD, Beasley WH, van den Oord EJCG (2019) Responding to a 100-year-old challenge from fisher: a biometrical analysis of adult height in the NLSY data using only cousin pairs. Behav Genet 49(5):444–454. 10.1007/s10519-019-09967-6 31392459 10.1007/s10519-019-09967-6 PMC6778682

[CR53] Satorra A, Saris WE (1985) Power of the likelihood ratio test in covariance structure analysis. Psychometrika 50(1):83–90. 10.1007/BF02294150

[CR54] Schaack S, Ho EKH, Macrae F (2019) Disentangling the intertwined roles of mutation, selection and drift in the mitochondrial genome. Philos Trans R Soc B Biol Sci 375(1790):20190173. 10.1098/rstb.2019.0173 10.1098/rstb.2019.0173 PMC6939366 31787045

[CR55] Sham PC, Purcell SM, Cherny SS, Neale MC, Neale BM (2020) Statistical power and the classical twin design. Twin Res Hum Genet 23(2):87–89. 10.1017/thg.2020.46 32638684 10.1017/thg.2020.46

[CR56] Smith KR, Fraser A, Reed DL, Barlow J, Hanson HA, West J, Mineau GP (2022) The utah population database: a model for linking medical and genealogical records for population health research. Historical Life Course Stud 12:58–77. 10.51964/hlcs11681

[CR57] Verhulst B (2017) A power calculator for the classical twin design. Behav Genet 47(2):255–261. 10.1007/s10519-016-9828-9 27866285 10.1007/s10519-016-9828-9 PMC5471839

[CR58] Visscher PM (2004) Power of the classical twin design revisited. Twin Res 7(5):505–512. 10.1375/1369052042335250 15527666 10.1375/1369052042335250

[CR59] Visscher PM, Gordon S, Neale MC (2008) Power of the classical twin design revisited: II detection of common environmental variance. Twin Res Hum Genet 11(1):48–54. 10.1375/twin.11.1.48 18251675 10.1375/twin.11.1.48 PMC3996914

[CR60] Wang YA, Rhemtulla M (2021) Power analysis for parameter estimation in structural equation modeling: a discussion and tutorial. Adv Methods Pract Psychol Sci 4(1):2515245920918253. 10.1177/2515245920918253

[CR61] Winklhofer KF, Haass C (2010) Mitochondrial dysfunction in Parkinson’s disease. Biochimica et Biophysica Acta (BBA) - Mol Basis Dis 1802(1):29–44. 10.1016/j.bbadis.2009.08.013 10.1016/j.bbadis.2009.08.013 19733240

[CR62] Xing J, Chen M, Wood CG, Lin J, Spitz MR, Ma J, Wu X (2008) Mitochondrial dna content: its genetic heritability and association with renal cell carcinoma. JNCI J Natl Cancer Inst 100(15):1104–1112. 10.1093/jnci/djn213 18664653 10.1093/jnci/djn213 PMC2720693

